# Targeting SARS-CoV-2-Platelet Interactions in COVID-19 and Vaccine-Related Thrombosis

**DOI:** 10.3389/fphar.2021.708665

**Published:** 2021-07-05

**Authors:** Dermot Cox

**Affiliations:** School of Pharmacy and Biomolecular Sciences, Royal College of Surgeons in Ireland, Dublin, Ireland

**Keywords:** platelets, SARS-CoV-2, bacteria, sepsis, virus, immunothrombosis, Fcgamma receptor IIA, vaccine thrombosis

## Abstract

It is clear that COVID-19 is more than a pneumonia and is associated with a coagulopathy and multi-organ failure. While the use of anti-coagulants does reduce the incidence of pulmonary emboli, it does not help with survival. This suggests that the coagulopathy is more likely to be platelet-driven rather than thrombin-driven. There is significant evidence to suggest that SARS-CoV-2 virions directly interact with platelets to trigger activation leading to thrombocytopenia and thrombosis. I propose a model of multiple interactions between SARS-CoV-2 and platelets that has many similarities to that with *Staphylococcus aureus* and Dengue virus. As platelet activation and thrombosis are major factors in poor prognosis, therapeutics that target the platelet-SARS-CoV-2 interaction have potential in treating COVID-19 and other virus infections.

## Introduction

### COVID-19

During the month of December 2019, 41 patients presented to hospital in Wuhan, China with an unusual pneumonia of unknown aetiology. The common factor between these patients was a local ‘wet’ market. One month from the first case, and prior to any deaths, the pathogen was identified as a novel Coronavirus (SARS-CoV-2) and its genetic sequence made available. The initial view was that this was a pneumonia and that mortality was associated with acute respiratory distress syndrome (ARDS) (Arentz et al., 2020; Guan et al., 2020). Like SARS-CoV it was found to bind to angiotensin converting enzyme (ACE) 2, which is expressed on the surface of cells in the lungs, thereby facilitating entry into the cells (Hoffmann et al., 2020). However, it soon became clear that many of the deaths were due to coagulopathy and multi-organ failure that were associated with an increase in D-dimer levels (Tang et al., 2020b). This led to recommendations for use of heparin in managing COVID-19 patients (Tang et al., 2020a; Levi et al., 2020). This COVID-19 associated coagulopathy is accompanied by a thrombocytopenia that is a predictor of outcome (Yang et al., 2020).

### Infection and Thrombocytopenia

Thrombocytopenia is a common manifestation of serious infections such as sepsis (Ree et al., 2017) but also with some viral infections (Raadsen et al., 2021). As the magnitude of the thrombocytopenia is related to severity of the infection, as well as patient survival, there is clear evidence of an association between platelets and infection. There are a number of potential mechanisms behind this thrombocytopenia.

#### Platelet Sequestration

One possibility is that the thrombocytopenia is due to infection of the megakaryocytes resulting in a reduction in platelet synthesis. There is evidence that some viruses such as Dengue (DENV) and Influenza virus can infect megakaryocytes, and in turn trigger an anti-viral response (Campbell et al., 2019). Megakaryocyte infection by DENV can lead to reduced megakaryocyte levels, which would result in reduced platelet production (Vogt et al., 2019). As the lifespan of a platelet is around 10 days this would suggest that if platelet production was completely inhibited it would take 5 days for a 50% drop in platelet count. Furthermore, platelet activation or inflammation leads to increased levels of IL-1 and CCL5 which both increase platelet production by around 50% (Nishimura et al., 2015; Machlus et al., 2016) and evidence of increased platelet production has been found in COVID-19 (Roncati et al., 2020). Thus, while there is evidence that viruses can infect megakaryocytes this can result in both increased platelet production as well as the loss of some megakaryocytes. Therefore, as it is unlikely that there would be complete inhibition of platelet production, thrombocytopenia would occur slowly and probably not to the extent seen clinically.

#### Platelet Sequestration

A related mechanism for thrombocytopenia is sequestration of platelets in spleen, liver etc., however, it is unlikely that thrombocytopenia due to sequestration of platelets or reduced platelet synthesis would be of major clinical significance. While thrombocytopenia occurs when the platelet count falls below 100,000 platelets/μL this has only minor clinical consequences and a usual threshold for surgery is a count of 50,000 platelets/μL. Even then, spontaneous bleeding is unlikely to occur until the platelet count reaches 10,000 platelets/μL (Slichter, 2004). So if thrombocytopenia is simply due to a lack of platelets this might cause some nuisance bleeds but would not affect the disease process. While the presence of thrombocytopenia may be a biomarker for the disease severity, thrombocytopenia itself is unlikely to be involved in the pathogenesis.

#### Platelet Activation

The other possibility for thrombocytopenia is due to platelet activation and consumption. This has the potential to be more serious as activated platelets will form thrombi. These thrombi can occlude blood vessels in the liver, spleen etc., which can become occluded leading to ischemic damage. If thrombus formation occurs to a significant extent, it ultimately leads to organ failure. This multi-organ failure is characteristic of severe sepsis and severe viral infections. Thus, thrombocytopenia due to platelet activation is a serious event and likely directly drives the multi-organ failure.

One potential mechanism for platelet activation in response to infection is that of innocent by-stander. Infection triggers a systemic inflammatory response, which may lead to direct activation of platelets or to thrombin generation. There is evidence that inflammatory cytokines such as IL-6 and IL-8 can activate platelets (Lumadue et al., 1996). However, activated platelets are also an important source of pro-inflammatory cytokines (Singh et al., 2020). Inflammation and coagulation are directly linked in a bi-directional manner and thus inflammation leads to thrombin generation in a tissue factor-dependent manner (Levi et al., 2004). This thrombin generation leads to the formation of a fibrin clot but also leads to platelet activation and consumption. Excess thrombin generation can be neutralised by anti-thrombin agents. Activated Protein C, an endogenous inhibitor of thrombin, came on the market for the treatment of sepsis (Bernard et al., 2001) but was ultimately withdrawn due to lack of efficacy (Martí-Carvajal Arturo et al., 2012). Similarly, the use of other anti-thrombin agents has failed to impact on survival in sepsis (Umemura et al., 2016). All attempts to control the coagulopathy of sepsis by targeting thrombin generation have failed (Cavaillon et al., 2020). Thus, while thrombin generation does occur in sepsis, it is clearly not the driver of the thrombocytopenia.

#### Immunothrombosis

If platelet activation is not driven by thrombin generation, the other likely mechanism is direct interaction of pathogens with platelets. While usually thought of as key mediators of haemostasis there is growing awareness of the role of platelets in the innate immune response, which has led to the concept of immunothrombosis (Engelmann and Massberg, 2013). Infection is usually associated with a breach of the vasculature, and platelets, as the first responders to such damage, are in a good position to act as regulators of the subsequent immune response. To facilitate this, it is necessary for platelets to interact directly with pathogens so that they can respond appropriately to infection. It is no coincidence that most of the pathogens that trigger thrombocytopenia are also capable of binding to platelets and activating them (Fitzgerald et al., 2006a).

As an important role for platelets in innate immunity is the production of anti-microbial peptides (Yeaman, 2014), some pathogens have developed the ability to prevent platelet activation and thus reduce exposure to the anti-microbial peptides. *Yersinia pestis*, the causative agent of bubonic plague, has been shown to inhibit platelet activation (Palace et al., 2020). However, *Y. pestis* can cause infective endocarditis, which is caused by the formation of a thrombus on a heart valve (Ioannou et al., 2021) and is often associated with thrombocytopenia (Welty et al., 1985).

### Platelet Receptors

Platelets contain numerous receptors on their surface that enable them to respond to stimuli. We can consider that there are two types of receptors. There are those receptors that respond to stimuli and trigger the activation of platelets and those receptors that facilitate the haemostatic process.

The primary receptor that facilitates haemostasis is GPIIb/IIIa (integrin αIIbβ3) and is the most highly expressed receptor on the platelet surface. It is the receptor for fibrinogen, which is a dimer containing two *α*, *β* and *γ* chains. As a dimer, it can bind to two different GPIIb/IIIa molecules and if these are on different platelets, cross-links platelets leading to the formation of a thrombus. GPIIb/IIIa is not just a fibrinogen-binding protein but also a true receptor that generates signals that are important for reinforcing the initial activating signals (Coller, 2015).

The receptors for platelet activating signals can be divided into two groups–those that trigger the haemostatic properties of platelets and those that trigger the immune function of platelets. The ‘classic’ receptors for platelet activation include G-protein-coupled receptors (ADP receptor; P2Y12) protease-activated receptors (thrombin receptor), integrins (collagen receptor α2β1) and glycoproteins (von Willebrand factor receptor GPIb/IX/V). Activation of these receptors generate signals (outside-in) and subsequent inside-out signals that activate GPIIb/IIIa facilitating fibrinogen binding and thrombus formation. They also trigger granule release (α-granules, dense granules and lysosomes) (Stalker et al., 2012).

Aside from the receptors that mediate the haemostatic functions, platelets also express a distinct set of receptors that respond to pathogens and trigger the immune function of platelets such as FcγRIIa, Toll-like receptors and lectins. Many of these are primarily associated with immune cells, which underscores the role of platelets in the immune system. Furthermore, the response of the platelets is also different (Fitzgerald et al., 2006a; Cox, 2007).

### FcγRIIa

FcγRIIa is probably the best-studied immune receptor on platelets (Patel et al., 2021). It is a receptor for the Fc portion of IgG and is the most significant member of the FcγR family of receptors. All of the members of the FcγR family, with the exception of FcγRIIb, are stimulatory receptors due to the presence of the ITAM domain. FcγRIIb is an inhibitory receptor with an ITIM domain. FcγRIIa is the only Fc receptor on platelets (Hogarth and Pietersz, 2012).

FcγRIIa is usually associated with phagocytosis. Binding of immune complexes to FcγRIIa facilitates phagocytosis and thus it is present on cells such as monocytes/macrophages. Their presence on platelets is surprising as they are the only non-phagocytic cells that express FcγRIIa, although, while not true phagocytes, platelets have been shown to engulf pathogens (White, 2005; Gaertner et al., 2017). However, its role goes beyond phagocytosis. In monocytes/macrophages immune complex binding to FcγRIIa also triggers tumour necrosis factor (TNF)-α production, which mediates the inflammation in rheumatoid arthritis and Crohn’s disease. Immune complex engagement with FcγRIIa on platelets results in platelet activation and aggregation. These immune complexes are either complexes of agglutinated immunoglobulins, pathogen-IgG or platelet-IgG. FcγRIIa-oligomerisation is necessary to trigger platelet action and this can involve formation of homo or hetero-oligomers.

A typical example of homoligomerisation occurs with heat-agglutinated IgG. The addition of heat-agglutinated IgG to platelets results in aggregation due to cross-linking of FcγRIIa (Peerschke and Ghebrehiwet, 1997). This likely occurs with immune thrombocytopenia where platelets coated with IgG bind to FcγRIIa and trigger platelet activation (McKenzie et al., 1999).

Many bacteria have been shown to directly induce platelet aggregation in an FcγRIIa-dependent manner (Fitzgerald et al., 2006a). This aggregation response differs from aggregation induced by the ‘classic’ platelet agonists. With the ‘classic’ agonists, aggregation occurs immediately (within a few seconds) and the magnitude of the response is dependent on the concentration of agonist. However, bacteria-induced aggregation is different. Aggregation occurs after a delay (lag time). Rapid response occurs within a few minutes of adding bacteria while a slow response in excess of 15 min occurs with some bacteria. Some strains of bacteria do not induce aggregation at all (lag time greater than 30 min). The aggregation response with bacteria is all-or-nothing with either no aggregation or maximum aggregation occurring. Reducing the concentration of bacteria results in a prolongation of the lag time rather than a reduction in the extent of aggregation (Kerrigan et al., 2002).

Staphylococci induce aggregation with a short lag time that is dependent on binding of anti-Staphylococci antibodies, which in turn engages with FcγRIIa (Loughman et al., 2005). However, this is not sufficient to trigger aggregation. *S. aureus* also expresses fibrinogen-binding proteins such as clumping factors (Clf) A & B and fibronectin-binding proteins (Fnbp). This bound fibrinogen or fibronectin binds to platelet GPIIb/IIIa and is essential for the aggregation response (O'Brien et al., 2002). *S. epidermidis* expresses serine-aspartate repeat (Sdr) G, which can directly bind to GPIIb/IIIa inducing aggregation in an IgG and FcγRIIa-dependent manner (Brennan et al., 2009). Thus, Staphylococci act to crosslink FcγRIIa with GPIIb/IIIa to trigger platelet activation and aggregation.

Streptococci such as *S. sanguinis* (Kerrigan et al., 2002) and *S. gordonii* (Kerrigan et al., 2007) also induce platelet aggregation in an FcγRIIa-dependent manner. Streptococci express GPI-b binding proteins such as serine-rich protein (srp) A on *S. sanguinis* (Plummer et al., 2005) and Hsa on *S. gordonii* (Kerrigan et al., 2007). While IgG does not appear to be necessary for Streptococci-induced platelet aggregation, FcγRIIa is necessary (Kerrigan et al., 2002) and there is evidence that both FcγRIIa and GPIb are co-localised (Sullam et al., 1998). On the other hand, *Helicobacter pylori* express a von Willebrand factor-binding protein and also bind IgG, which induces platelet aggregation by crosslinking FcγRIIa and GPIb (Byrne et al., 2003). A similar effect can happen with *S. aureus* where protein A can bind von Willebrand factor and in the presence of anti-*S. aureus* antibody can induce platelet aggregation (O'Seaghdha et al., 2006). Thus, bacteria can induce platelet aggregation by crosslinking FcγRIIa and GPIb.

Even in the absence of a direct interaction of the bacteria with platelets, there is a more generic interaction with platelets. Bacteria can bind complement, which can interact with a complement receptor on the platelet surface and trigger platelet aggregation. This aggregation is slow (greater than 15 min lag time) but also requires IgG binding to FcγRIIa. An example of this is *Escherichia coli*-induced platelet aggregation (Moriarty et al., 2016; Watson et al., 2016). Furthermore, when the fibrinogen-binding proteins are deleted from *S. aureus* they can still induce aggregation in a complement-dependent manner although the lag time is significantly increased (Fitzgerald et al., 2006b). Another factor that can also lead to platelet aggregation is binding of PF4 to bacteria, which acts to enhance FcγRIIa-mediated aggregation (Arman et al., 2014).

Viruses can also induce platelet activation in an FcγRIIa-dependent manner. Dengue virus (DENV) can trigger massive platelet activation, which leads to Dengue haemorrhagic fever (DHF). There are five serotypes of DENV and infection with any one serotype produces only minimal effects such as flu-like symptoms. However, infection with a second serotype can lead to DHF. This is due to a phenomenon known as antibody-dependent enhancement (ADE). After exposure to the first serotype, the patient produces inhibitory antibodies and is fully immune from infection with that serotype. However, if they are exposed to a second serotype they have pre-existing, cross-reacting, non-inhibitory antibodies, which produces virus particles coated in non-inhibitory antibody. In a process similar to that with *S. aureus* these antibody-coated virions cause massive platelet activation in an FcγRIIa-dependent manner (Simmons et al., 2012; Screaton et al., 2015). Influenza H1N1 (Boilard et al., 2014) and some Bunyaviruses such as Crimea-Congo Haemorrhagic fever (Erduran et al., 2013), also induce platelet activation *via* FcγRIIa.

Thus, FcγRIIa plays an important role in mediating platelet activation by bacteria and viruses, usually in an IgG-dependent manner. Therefore, it is not surprising that the major causative agents of sepsis are commensals such as Staphylococci, Streptococci and *E. coli* as everybody has antibodies to these pathogens. Typically, bacteria that activate platelets also have a secondary mechanism for interacting with platelets by binding to platelet receptors such as GPIIb/IIIa, GPIb or complement receptor, either directly or by binding a bridging molecule such as fibrinogen or von Willebrand factor.

### Toll-Like Receptors

FcγRIIa is not the only immune receptor expressed on platelets as they also express TLRs (Hally et al., 2020). In terms of platelet function the two most important TLRs are TLR2 and TLR4, however, the functionality of these receptors has been disputed (Cognasse et al., 2015). Lipopolysaccharide (LPS) is a TLR4 agonist and LPS from *E. coli* O157 fails to induce platelet aggregation even though *E. coli* O157 can induce aggregation (Moriarty et al., 2016). However, the TLR2 agonist Pam3Csk4 can induce platelet aggregation in a TLR2-dependent manner (Moriarty et al., 2016) while *Streptococcus pneumoniae* (Keane et al., 2010) and cytomegalovirus (Assinger et al., 2014) also induce platelet aggregation in a TLR2-dependent manner. Encephalomyocarditis virus has been shown to bind to and activate platelets in a TLR7-dependent manner (Koupenova et al., 2014). A key role for pathogen-binding to platelet TLR is to facilitate the platelet-leucocyte interaction (Dib et al., 2020; Marín Oyarzún et al., 2020) and it may play a role in endocytosis of virions by platelets (Banerjee et al., 2020).

### Other Immune Receptors

Pathogens, especially viruses, have also been shown to interact with other immune receptors on platelets (Assinger, 2014). Lectins are a family of receptors that recognise carbohydrates but can also bind to proteins. C-type lectins are calcium-dependent lectins and their role in binding viruses means they are recognised as pathogen-recognition receptors (Rahimi, 2020). There are a number of C-type lectins found on platelets including DC-SIGN (Dendritic Cell-Specific Intercellular adhesion molecule-3-Grabbing Non-integrin) and CLEC (C-type lectin-like receptor) 2. DC-SIGN, which binds HIV (Chaipan et al., 2006), DENV (Tomo et al., 2018), Ebola virus (Alvarez et al., 2002), Hepatitis virus (Pöhlmann et al., 2003) and influenza H1N1 (Londrigan et al., 2011) and H5N1 (Yang et al., 2021), is implicated in platelet activation. CLEC-2 and 5A are also important in binding to viruses (Sung and Hsieh, 2019) such as HIV (Chaipan et al., 2006).

### Pathogen-Induced Thrombosis

It is clear that platelets can interact with numerous bacteria and viruses as part of the normal host response to infection. Furthermore, pathogens such as Plasmodium (malaria) can also cause platelet activation [for review (Cox and McConkey, 2010)]. The purpose of these interactions is to contain the infection through direct killing of the pathogen and recruitment of other immune cells to the site of infection. However, this does not always work, and if the pathogen does not die there can be on-going platelet activation leading to thrombotic events. While every pathogen is unique, there are some common factors to the pathogen-platelet interactions. FcγRIIa is the primary receptor for mediating platelet-pathogen interactions and this usually involves anti-pathogen antibodies. Pathogens that can assemble complement can also activate platelets. TLRs and lectins are other platelet receptors that interact with pathogens. These general principles are a good starting point for investigating thrombocytopenia to a novel pathogen such as SARS-CoV-2.

### Covid-19 and Thrombosis

With the initial SARS-CoV-2 cases, it was thought that this was a typical respiratory viral infection (Guan et al., 2020; Jiang et al., 2020) that could in some cases lead to pneumonia or even acute respiratory distress syndrome (ARDS). While infected patients did die of pulmonary complications it was distinct from ARDS (Archer et al., 2020) and it soon became clear that many patients who died had developed multi-organ failure (Mokhtari et al., 2020), i.e., they had developed a viral sepsis.

Not surprisingly, it also became clear that patients with Covid-19 developed a coagulopathy (Zhang L. et al., 2020; Chen et al., 2020; Trimaille et al., 2020) along with thrombocytopenia. However, COVID-19-associated coagulopathy is distinct from that of influenza-associated coagulopathy (Zhang et al., 2021). Initial focus was on thrombin generation due to the increase in D-dimer levels, which was associated with mortality (Abou-Ismail et al., 2020; Zhang L. et al., 2020), and led to the use of heparin in the management of Covid-19 (Tang et al., 2020a; Thachil et al., 2020). A role for increased levels of Tissue Factor has been proposed (Bautista-Vargas et al., 2020). Initial recommendations were for use of prophylactic doses of low molecular weight heparin (Thachil et al., 2020). However, it soon became clear that patients continued to have thrombotic events on prophylactic heparin (Trimaille et al., 2020) and some studies suggested that using intermediate dose heparin was more effective (Stessel et al., 2020; Meizlish et al., 2021a) although other studies showed no benefit from the increased dose of heparin (Investigators, 2021). One study found that therapeutic anti-coagulation in COVID-19 patients reduced the risk of pulmonary embolism but had no effect on mortality (Spiegelenberg et al., 2021).

This is reminiscent of bacterial sepsis where anti-coagulants such as activated protein C and heparin provided some benefit but never really resolved the issue. As with bacterial sepsis, this suggests that thrombin generation is only partly responsible for the coagulopathy and that direct activation of platelets by pathogen is more likely to be responsible for the coagulopathy.

#### COVID-19 Thrombocytopenia

Thrombocytopenia has been well established with Covid-19 (Bomhof et al., 2020) and the extent of thrombocytopenia is associated with outcome (Liu Y. et al., 2020; Lippi et al., 2020; Yang et al., 2020). The presence of micro-thrombi in coronary vessels of Covid-19 patients has been confirmed after autopsy (Rapkiewicz et al., 2020; Pellegrini et al., 2021). Evidence also suggests that pulmonary thrombosis arises within the lung rather than as a DVT embolism (Gabrielli et al., 2020). SARS-CoV-2 RNA has been found in platelets and infection is associated with platelet activation and degranulation (Zaid et al., 2020). Further evidence of platelet activation is the increased levels of platelet microparticles in Covid-19 patients (Cappellano et al., 2021).

#### SARS-CoV-2-Platelet Interactions

There is evidence that SARS-CoV-2 can interact with platelets. Interaction of pathogens with platelets is mediated either by a direct interaction of a pathogen surface protein with a platelet receptor or by pathogen-bound antibody binding to FcγRIIa. SARS-CoV-2 has only four structural proteins–spike (S), nucleocapsid (N), membrane (M) and envelope (E) proteins. The spike protein of Corona viruses including SARS-CoV-2 binds to ACE2 receptor *via* its receptor-binding domain (RBD) (Walls et al., 2020). ACE2 has been found to be expressed on platelets and mediates SARS-CoV-2 binding (Zhang S. et al., 2020). Both SARS-CoV (Shih et al., 2006) and SARS-CoV-2 (Amraei et al., 2020) spike proteins bind to DC-SIGN, a protein expressed on platelets. The spike protein also contains an Arg-Gly-Asp (RGD) sequence that would potentially allow it to interact with integrins (Makowski et al., 2021; Mészáros et al., 2021). GPIIb/IIIa is the primary receptor on the platelet surface and it binds multiple RGD-containing ligands. As the ability to bind GPIIb/IIIa has been shown to be critical for *S. aureus*-induced platelet activation the same may be true of SARS-CoV-2. SARS-CoV-2 envelope protein has been shown to interact with TLR-2 (Zheng et al., 2021), which is found on platelets and mediates platelet activation in response to *S. pneumoniae* (Keane et al., 2010).

#### Role of Antibody

There is also the potential for a role for antibody production. Within 1 week of the onset of symptoms of COVID-19, over 50% of patients had significant anti-SARS-CoV-2 IgG levels that were higher in patients with severe disease (Long et al., 2020) and this reflects the time to disease progression as most patients are admitted to ICU during the second week after the on-set of symptoms (Zhou et al., 2020). Studies of antibody production post-exposure to SARS-CoV-2 and subsequent clinical outcome found that patients with mild disease had lower titres of antibodies although the ratio of anti-RBD to anti-N was higher (Atyeo et al., 2020; Röltgen et al., 2020). Furthermore, patients who died had higher levels of anti-N antibodies that fixed complement (Atyeo et al., 2020). The glycosylation profile of the anti-SARS-CoV-2 IgG also appears to be important with a low fucosylated form being more pro-inflammatory (Hoepel et al., 2021) and more pro-thrombotic (Bye et al., 2021). Covid-19 is associated with increased complement formation (Fletcher-Sandersjöö and Bellander, 2020; Noris et al., 2020) and complement fragments have been found in coronary micro-thrombi after autopsy, and SARS-CoV-2 can directly activate complement formation *via* the alternative pathway (Yu et al., 2020). The fact that sicker patients have higher antibody titres and higher levels of platelet activation may suggest a potential role for antibody in the interaction of SARS-CoV-2 with platelets especially as these antibodies have increased potential to fix complement, which has been shown to be important in bacteria-induced platelet aggregation. These antibodies may be pre-existing as SARS-CoV-2 can generate antibodies that cross-react with other Corona viruses (Ladner et al., 2021). Antibodies to spike protein have been found to lead to platelet activation in COVID-19 patients that was FcγRIIa-dependent (Nazy et al., 2021a). Both FcγRIIa signalling and complement formation have been found to mediate platelet activation by SARS-CoV-2 (Apostolidis et al., 2021).

#### Role of Neutrophils

Neutrophil activation has been shown to occur in Covid-19 (Veras et al., 2020; Meizlish et al., 2021b) and platelet activation is important for neutrophil activation and NET formation (Clark et al., 2007). This immunothrombosis is important in the pathogenesis of Covid-19 (Bonaventura et al., 2021).

### Targeting Platelets in COVID-19

If virus-induced platelet activation in important in the pathogenesis of Covid-19 then targeting platelets would be expected to improve survival. One retrospective study looked at the use of LMWH and aspirin in COVID-19 patients. They found that aspirin use was associated with a significant reduction in death (HR = 0.5) (Meizlish et al., 2021a). Interestingly, these patients were not taking aspirin for any underlying disease and were only given aspirin as part of their treatment for COVID-19. Another retrospective study found that aspirin use on admission or within 7-days of admission was associated with decreased mortality in COVID-19 (HR: 0.53) (Chow et al., 2021). Another retrospective study looked at aspirin use either pre-admission (60%) or after admission and found increased mortality in the aspirin group (32% *vs*. 22%) however, when they controlled for underlying risk factors they found a significant reduction in mortality in patients on aspirin (HR: 0.75) (Aghajani et al., 2021).

A large retrospective study showed that use of COX inhibitors (aspirin, ibuprofen, naproxen, ketorolac) or paracetamol was significantly associated with more severe Covid-19 but only aspirin and paracetamol were associated with increased mortality (Reese et al., 2021). Another study found no effect on mortality with aspirin or NSAIDS but did find an increase in the combined endpoint of MI, stroke or venous thromboembolism (Sahai et al., 2020). These results are unexpected and at odds with other studies. The discrepancies probably lie with the nature of the underlying disease and the NSAID being used. Paracetamol has no anti-platelet activity and thus its effects on mortality are unrelated to platelets. The timing of dosing is critical for benefit. In the studies that showed benefit for aspirin it was administered at admission or within 7-days. Patients who are on aspirin for fever and pain usually take it as needed. Thus, patients with migraine take it only when they have a migraine and unless these patients had a migraine 1 or 2 days before developing COVID-19 they may not have a current exposure to aspirin. Were these patients prescribed aspirin once admitted? Furthermore, patients on aspirin for secondary prevention for MI often have high levels of non-response due to the use of enteric-coated aspirin in patients with BMI in excess of 32 (McCall et al., 2020), which was the case in these studies, and also can have high levels of non-compliance (at least 14%) (Peace et al., 2010). In addition, the conditions for which many of these patients were being treated are associated with high levels of inflammation and are also high risk for severe COVID-19. Thus, it may be difficult to control for these risk factors.

Aspirin acts to inhibit the cyclooxygenase-dependent pathway of platelet aggregation and prevent the production of thromboxane A2. However, some agonists such as thrombin can activate platelets in a cyclooxygenase-independent manner. Many bacteria induce platelet aggregation in an aspirin-sensitive manner however, bacteria such as *S. pneumonia*e, which interacts with TLR-2, can activate platelets in an aspirin-independent manner (Keane et al., 2010). Thus, it is possible that SARS-CoV-2 activates platelets in an aspirin-independent manner. In this case a better anti-platelet agent to use would be a P2Y12 (ADP receptor) antagonist such as clopidogrel, prasugrel or ticagrelor. Clearly, there is sufficient evidence from retrospective studies to conduct a prospective study to investigate the benefit of anti-platelet therapy in COVID-19.

Why some individuals develop this highly thrombotic form rather than asymptomatic diseases is not clear but likely depends on a multitude of host and virus factors (DeMerle et al., 2021). Virus factors include the exposure dose and the SARS-CoV-2 variant. There are a number of clear risk factors for developing severe disease including age, obesity, diabetes and cardiovascular disease. One possible explanation is that these disorders are all associated with hyperactive platelets, which may be primed to respond to the virus (Iyer and Dayal, 2020). On the other hand pre-existing antibodies to some strains of Corona virus may protect from severe disease. Other host factors include polymorphisms in key receptors that bind the virus. There is a significant amount of work to be done to allow the prediction of patients at highest risk and thus requiring early treatment with anti-platelet agents.

### SARS-CoV-2-Associated Illnesses

While COVID-19 is the most common clinical presentation of SARS-CoV-2 infection, there are other clinical presentations, especially in children.

As SARS-CoV-2 binds to and inhibits ACE2 there are concerns that it may have cardiovascular implications through disruption of the renin-angiotensin system. These concerns are also reflected in the fact that pre-existing heart disease is considered a risk factor for severe disease. In contrast, data suggests that less people presented to hospital with acute myocardial infarction (De Filippo et al., 2020; Solomon et al., 2020) during the initial COVID-19 outbreak. However, this may be explained by increased out-of-hospital deaths from MI (Jain et al., 2021). In fact, the COVID-19 death rate is much higher than anticipated as a recent study found a 22% excess mortality in the United States from March to December 2020. While 75% of this was due to COVID-19 there was a 5% increase in cardiovascular deaths (Woolf et al., 2021). As a pro-thrombotic disorder, it would be expected that there would be an increase in the incidence of myocardial infarction (MI) in SARS-CoV-2 infected patients although there is no identifiable lesion in around 50% of cases (Lang et al., 2020; Stefanini et al., 2020). Patients often present with ST-elevated MI (STEMI) (Stefanini et al., 2020). This association between infection and MI is not unique to SARS-CoV-2 as sepsis is also associated with an increase in MI.

While children are at a very low risk from developing severe Covid-19 there are some unique rare complications of Covid-19 in children (Williams et al., 2020). Kawasaki disease is a rare complication associated with SARS-CoV-2 infection in children (Esper et al., 2005; Verdoni et al., 2020) although there have been cases in adults as well (Shaigany et al., 2020). Kawasaki disease is a vasculitis that usually affects young children and is the most common acquired heart disease in children (McCrindle et al., 2017). Kawasaki disease is associated with platelet activation (Alam, 2017). Recommended treatment is IVIg, an inhibitor of FcγRIIa, and aspirin (McCrindle et al., 2017). One effect of this treatment is inhibition of immune complex activation of platelets.

Another rare presentation of SARS-CoV-2 in children is a Kawasaki-like syndrome known as Multisystem Inflammatory Syndrome in Children (MIS-C). As its name implies this syndrome affects children and is associated with fever, evidence of inflammation, involvement of at least two organs and infection with SARS-CoV-2. It is a diagnosis of exclusion. It has been associated with heart failure (Belhadjer et al., 2020). IVIg has been shown to be beneficial in MIS-C (Ouldali et al., 2021).

### Vaccine-Related Thrombosis

Despite all of the odds, one year after the first case of Covid-19 in Wuhan, China, the first vaccine received emergency authorisation. Since then, a number of vaccines have been approved using two different technologies. The first to be approved used a novel mRNA-based vaccine and was used by both Pfizer and Moderna. Subsequently, vaccines based on conventional technology using Adenovirus vectors, were approved (Astra Zeneca and J&J). However, recently there have been concerns about a risk of thrombosis with the Adenovirus vaccines.

Initial reports from Norway (Schultz et al., 2021) and other European countries (Greinacher et al., 2021; Scully et al., 2021) suggested that the Astra Zeneca vaccine was associated with an unusual thrombotic event. In Norway, vaccine distribution was halted after five patients developed severe thrombocytopenia. As only 130,000 people had been vaccinated at this stage this represented an incidence of 1:25,000. All developed symptoms within 10 days of vaccination. All developed thrombocytopenia with platelet counts as low as 10,000 platelets/μL and had thrombotic events in unusual places such as the cortical vein, inferior sagittal sinus or hepatic vein. There was also a case of a woman who developed a stroke due to a thrombus in the carotid artery (Blauenfeldt et al., 2021). Some also developed cerebral haemorrhage as well as the thrombotic events. Many of the patients were give IVIg although this did not resolve the issue for all patients. IVIg is an inhibitor of FcγRIIa.

One thing in common with most of these patients was the presence of anti-PF4 antibodies despite no history of heparin use. In fact, platelet activation was heparin-independent, unlike that seen in heparin-induced thrombocytopenia (HIT), although activation was inhibited by heparin (Nazy et al., 2021a). While anti-PF4 antibodies are responsible for mediating HIT and are common in patients with sepsis (Maharaj and Chang, 2018) the majority of anti-PF4 antibodies do not induce platelet activation (Nazi et al., 2015). An important treatment for HIT is intravenous IgG (IVIg), which is known to work by inhibiting FcγRIIa. IVIg has also been used for patients with vaccine-mediated thrombosis. PF4 has been shown to bind bacteria and facilitate phagocytosis (Krauel et al., 2011) and also enhance platelet activation (Arman et al., 2014). PF4 secreted by platelets has been shown to enhance monocyte replication of DENV and Japanese encephalovirus (Ojha et al., 2019). At low concentrations, PF4 binding to HIV inhibits infection while at physiological concentrations it enhances infection by enhancing virus attachment (Parker et al., 2016). It also plays a protective role in influenza H1N1 infection (Guo et al., 2017). Increased levels of PF4 and anti-PF4 antibodies are found in patients with Covid-19 (Liu X. et al., 2020; Cai et al., 2020) and there is a report of a patient who developed immune thrombocytopenia after SARS-CoV-2 infection (Zulfiqar et al., 2020).

Thus, vaccine-mediated thrombosis is due to immune complex formation, although the nature of these complexes is unclear. Patients with vaccine-mediated thrombosis have anti-spike protein antibodies (Nazy et al., 2021a) that may play a role especially as heparin blocks the thrombosis and heparin binds to spike protein RBD. An important treatment for immune complex diseases such as HIT is intravenous IgG (IVIg), which is known to work by inhibiting FcγRIIa. IVIg has been used for patients with vaccine-mediated thrombosis (Bourguignon et al., 2021). Both anti-platelet agents and FcγRIIa inhibition have also been shown to prevent platelet activation mediated by serum from patients with vaccine-induced thrombosis (Smith et al., 2021).

Thrombocytopenia is reported to be a common (1–10% incidence) occurrence with AZD1222 although thrombosis is considered to be a very rare event. A recent Scottish study investigated the incidence of adverse events after COVID-19 vaccination. They found that there was an excess (observed-expected) of 1.33 cases of non-immune thrombocytopenia per 100,000 people and 0.45 per 100,000 for immune thrombocytopenia. There was also evidence of a small increase in arterial thrombosis and haemorrhagic events but no increase in venous thromboembolic events (Simpson et al., 2021). Thus, it would appear that platelet activation in response to AZD1222 is a real but rare (around 1 in 100,000) event although it is not clear if this is due to a response to the Adenovirus vector or to the SARS-CoV-2 spike protein or even to a combination of the two.

Adenovirus vaccine vectors have previously been shown to interact with and activate platelets and these activated platelets are cleared form the circulation by Kupfer cells in the liver (Stone et al., 2007). This activation is mediated by a direct interaction with platelets although the platelet receptor is unknown (Gupalo et al., 2013). Adenoviruses are also used for gene therapy and onasemnogene abeparvovec was found to be associated with an asymptomatic thrombocytopenia (Friese et al., 2021). In mice, the interaction of Adenovirus with platelets is mediated by Coxsackie and Adenovirus receptor (CAR) and involves von Willebrand factor and p-selectin (Othman et al., 2007; Gupalo et al., 2011). Patients with vaccine-related thrombosis also have increased von Willebrand factor levels (Nazy et al., 2021a).

There have also been some cases of thrombocytopenia after the mRNA vaccines from Pfizer and Moderna (Lee et al., 2021; Julian et al., 2021), while others found no excess in cases of immune thrombocytopenia (Simpson et al., 2021; Welsh et al., 2021). As thrombocytopenia is rare, it is not clear if this represents the natural occurrence of thrombocytopenia or is related to the vaccine.

It is interesting that both SARS-CoV-2 infection and vaccination are both associated with thrombosis although there are some differences. Both show evidence of significant platelet activation although the clinical presentation differs. While both produce severe thrombocytopenia, in the case of infection this leads to multi-organ failure while vaccination leads to a more focal thrombosis typically in the brain or liver. This is not unique, as *S. aureus* infection causes thrombocytopenia and thrombosis that can present as sepsis or infective endocarditis–thrombus formation on heart valve (Sy et al., 2008). However, in both cases the mechanism of platelet activation is the same. The reason for thrombus formation occurring in one site over another is not always clear.

Both infection and vaccination are associated with a PF4-mediated thrombocytopenia. However, PF4 is stored in platelet granules and released upon activation. Thus, it is likely that in both cases there must be an initial platelet activation. Experience with multiple Adenoviruses suggest that AZD1222 initially interacts with platelets with some patients and activates them causing the release of PF4 along with other bioactive molecules. In the vast majority of cases where thrombocytopenia occurs this is a transient response as the virus does not replicate, however in a small number of cases a more prolonged platelet activation occurs leading to a thrombotic event.

It is not clear why some people develop this rare thrombotic event. One possible explanation may be found in Dengue haemorrhagic fever. Here the presence of non-inhibitory, cross-reacting antibodies mediate the extensive thrombocytopenia leading to DHF. Similarly, cross-reacting, non-inhibitory antibodies could be responsible for mediating SARS-CoV-2 vaccine thrombosis. Certainly, many COVID-19 patients have antibodies to other Coronaviruses. Antibodies to nucleocapsid protein of Coronavirus OC43 were protective for severe disease in in-patients although not out-patients. Antibodies to other strains were not protective (Dugas et al., 2021). Thus, pre-existing antibodies to a related Coronavirus or Adenovirus protein may be involved. In all cases of thrombosis the event happened within 10-days of vaccination, which is a very short time to generate an antibody response as in studies of SARS-CoV-2 infected patients it took 14-days to generate detectable antibody titres (Röltgen et al., 2020). Furthermore, it is possible that in some patients the vaccine initially reactivates memory B-cells directed against other Coronaviruses. Within 14 days, an immune response to the vaccine generates neutralising anti-RBD antibodies, however, if the patient was exposed to SARS-CoV-2 after vaccination but before the neutralising antibodies are produced, the non-neutralising antibodies may trigger a more robust immune response leading to platelet activation and thrombosis.

The question is how best to prevent and treat vaccine-related thrombosis? Due to its rarity there is no real benefit it actively trying to prevent it. However, after immunisation people are usually advised to take paracetamol to deal with some of the immediate adverse effects. A better approach might be to take an aspirin as not only will it prevent some of the immediate adverse effects such as fever, it may also help prevent thrombosis, although to be truly effective it may need to be administered for a few days. Management of vaccine-associated thrombosis has focused on the use of direct-acting anti-thrombin agents supplemented with IVIg but the use of aspirin and/or a P2Y12 antagonists such as ticagrelor or clopidogrel is like to be more effective.

### Platelets as a Drug Target in Infections

Thrombocytopenia is a common response to many pathogens. The evidence is clear that thrombocytopenia is not a benign event but evidence of platelet activation and consumption. This platelet activation causes thrombosis ultimately leading to multi-organ failure. All of the major pandemics are associated with platelet activation. Bubonic plague is associated with thrombocytopenia (Welty et al., 1985). The 1918 influenza outbreak was associated with haemorrhage, suggesting an involvement of platelets (Kobasa et al., 2007). Viral haemorrhagic fevers are associated with thrombocytopenia (Zapata et al., 2014) as is SARS-CoV-2. While the use of aspirin or P2Y12 antagonists may help prevent complications due to infection, a more targeted approach may be necessary. Targeting the pathogen is problematic as each pathogen has its own mechanism for interacting with platelets and thus any drug would be pathogen specific. On the other hand, there are a very limited number of receptors on platelets that are involved in the interaction with pathogens. Two receptors that are frequently involved are FcγRIIa and DC-SIGN. FcγRIIa is involved with platelet activation by many bacteria and viruses and would be an obvious drug target especially as small molecule antagonist have already been discovered (Hogarth and Pietersz, 2012). DC-SIGN has been shown to mediate the interaction between some viruses and platelets and would be a useful drug target for virus infections (Anderluh et al., 2012). The major advantage of using these agents is that the do not require identification of the pathogen which is important for a disease outbreak of a novel pathogen or in a bioterrorist attack (Cox et al., 2013). Furthermore, as these agents do not affect the thrombotic function of platelets they will not increase the bleeding risk.

An alternative approach is to target the signalling pathways of these receptors. Kinases such as Src, Syk and Btk mediate FcγRIIa-induced platelet activation and inhibitors of these kinases have been shown to prevent platelet activation by serum from a patient with vaccine-mediated thrombosis (Smith et al., 2021) and with COVID-19 (Apostolidis et al., 2021). Inhibitors of these kinases such as dasatinib (Src inhibitor), cerdulatinib (Syk inhibitor) and ibrutinib (Btk inhibitor) are already on the market as anti-cancer agents and the Syk inhibitor fostamatinib is approved for the treatment of immune thrombocytopenia (Connell and Berliner, 2019). While there are significant adverse effects associated with their use, there may be a role in an acute situation such as COVID-19. As COVID-19 is due to a hyper-immune response there has been interest in using immunosuppressant therapies in its management. Steroids have been found to be effective (Sterne et al., 2020) and there has been work on targeting specific cytokines such as IL-1 and IL-6 (Della-Torre et al., 2021; Eşkazan et al., 2021). The problem with using anti-cytokine therapy is that COVID-19 is associated with increases in multiple pro-inflammatory cytokines (Zaid et al., 2021) and it is not clear which one(s) to target. The benefit to targeting the platelet-virus interaction is that it can help prevent the increase in all pro-inflammatory cytokines.

## Conclusion

While initially considered a pulmonary disorder it has become clear that COVID-19 is a multi-system disorder and platelet activation is a major factor in this disease. While the mechanism of platelet activation is not known, it is possible to draw upon experiences with other infectious diseases. Considering that severe COVID-19 has similarities to bacterial sepsis this can help provide insight into likely mechanisms.

**FIGURE 1:**
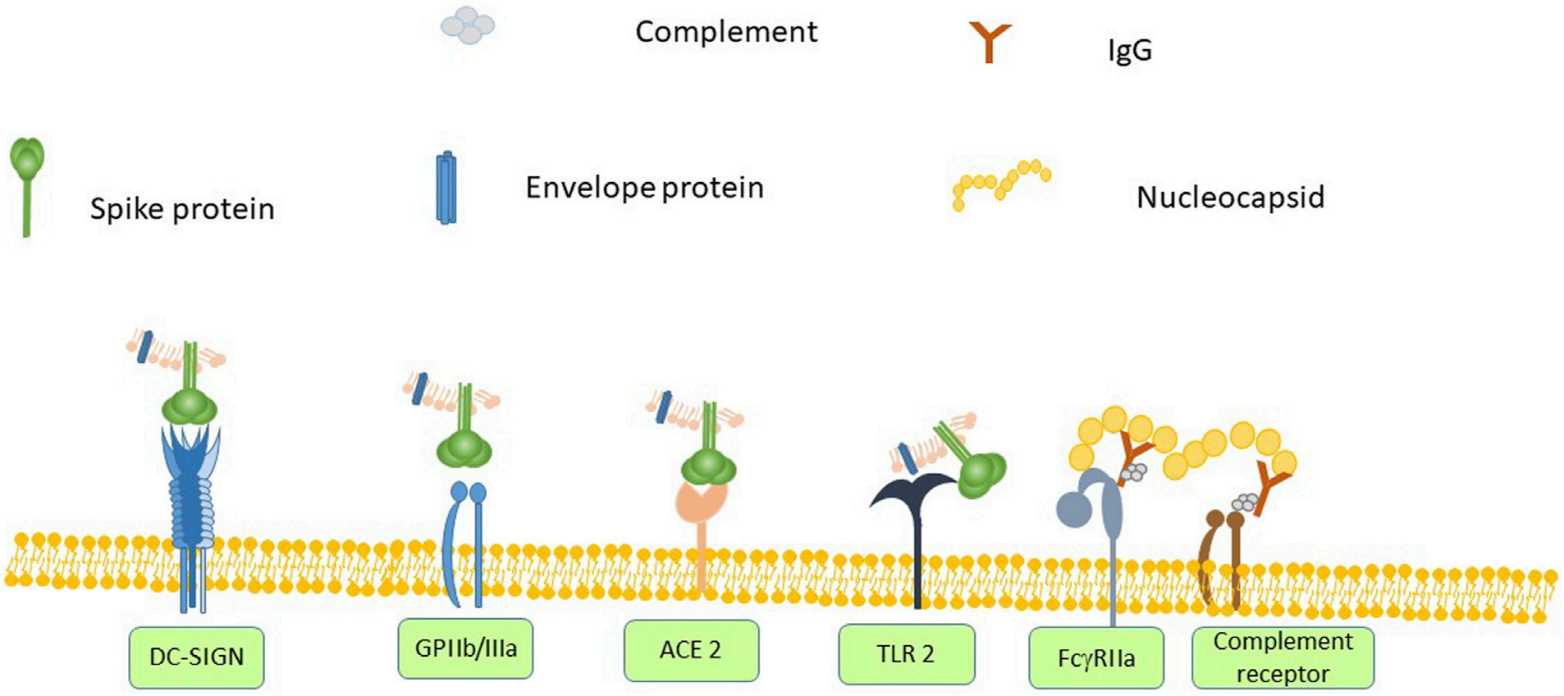
SARS-CoV-2 spike protein binds to angiotensin converting enzyme (ACE) 2 on any cell that expresses it, which includes platelets. Other platelet receptors can also interact with SARS-CoV-2 virions. Spike protein contains the amino acid sequence RGD, which would allow it to bind to GPIIb/IIIa and it also binds to DC-SIGN. The envelope protein can bind to Toll-like receptor (TLR)-2. Anti-SARS-CoV-2 antibodies can bind to FcγRIIa. Anti-nucleoprotein antibodies can also assemble complement, which in turn binds to a complement receptor on platelets. The combination of activating all of these receptors leads to platelet activation, secretion and ultimately thrombus formation.

Based on what is known about bacteria-platelet interactions in sepsis and Covid-19 there is a (see Figure 1) plausible model for SARS-CoV-2 –mediated platelet activation. SARS-CoV-2 spike protein binds to the ACE2 receptor and DC-SIGN on platelets and also has the potential to bind to GPIIb/IIIa *via* its RGD domain. The envelope protein can also bind to TLR2. Furthermore, binding of anti-nucleocapsid antibodies allows the fixation of complement. These antibody-coated virions can interact with platelet FcγRIIa and a platelet complement receptor. The resulting platelet activation releases many vasoactive compounds such as ADP and PF4. These further enhance the platelet activation and in the presence of pre-existing anti-PF4 antibodies can lead to a heparin-induced thrombocytopenia-like disorder. These interactions are sufficient to trigger wide-spread platelet activation leading to thrombocytopenia and multi-vessel thrombosis.

This platelet activation can be attenuated by the use of aspirin and it is likely that ADP-receptor antagonists such as clopidogrel, prasugrel or ticagrelor would also be effective in preventing COVID-19-associated thrombocytopenia. It is likely that an FcγRIIa-targeted therapy such as IVIg would also be effective in preventing the thrombocytopenia.

Platelets are a critical component of the innate immune system and play an important role in controlling infection. However, platelets can become part of the problem if they fail to control the infection but continue to be activated. In such case, pharmacological control of the platelets is necessary to prevent the consequences of uncontrolled platelet activation. This is not unique to SARS-CoV-2 and many pathogens have a similar effect. Targeting platelets has the potential to be an important strategy for managing serious complications of infection.
